# Supplementary material for: Genetic analysis of coronary artery disease using tree-based automated machine learning informed by biology-based feature selection
Source: IEEE/ACM Trans Comput Biol Bioinform. Author manuscript; Available in PMC 2022 Jul 18. (PMC9291719; doi:10.1109/TCBB.2021.3099068)
Supplement: supplemental — Fig. 1. Outline of the best pipeline from the resAdj TPOT stage 2 runs. [file NIHMS1813117-supplement-supplemental.pptx]

## Slide 1
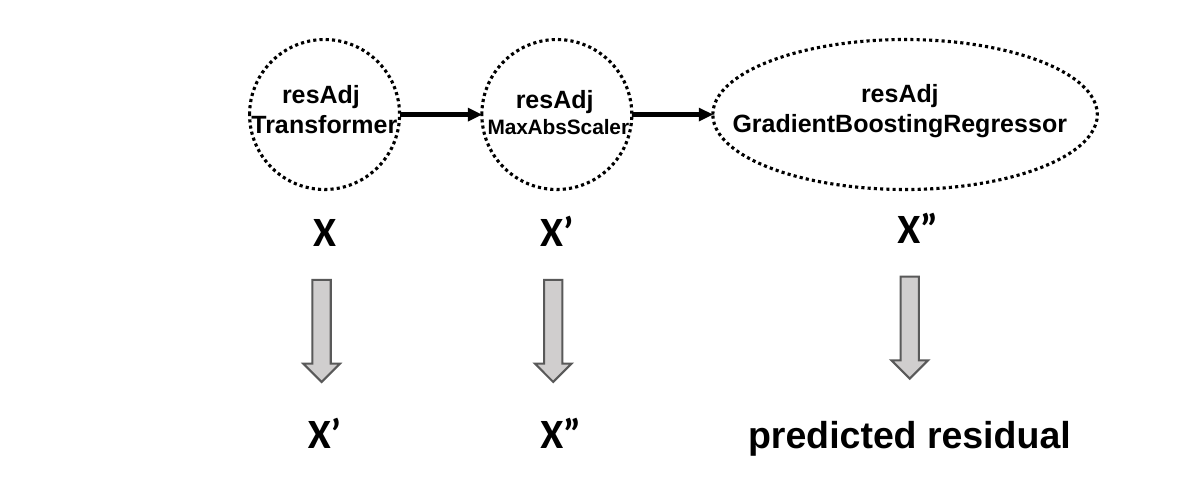

resAdj
Transformer
resAdj
MaxAbsScaler
resAdj
GradientBoostingRegressor
X”
X
X’
X’
X”
predicted residual
