# Supplementary material for: Genetic analysis of coronary artery disease using tree-based automated machine learning informed by biology-based feature selection
Source: IEEE/ACM Trans Comput Biol Bioinform. Author manuscript; Available in PMC 2022 Jul 18. (PMC9291719; doi:10.1109/TCBB.2021.3099068)
Supplement: supplemental table 1 — SNPs in the PPP2R3A-PRC1 feature set. For each SNP, its CADD, GWAVA and TraP scores are indicated together with the gene the SNP maps to and whether it resides within the extended gene body (B) or a putative enhancer (E). [file NIHMS1813117-supplement-supplemental_table_1.pdf]

SNP	CADD	GWAVA	TraP	Gene	Region
rs188650245	10.6	NA	NA	PPP2R3A	B
rs77964675	10.46	NA	NA	PPP2R3A	B
rs139138366	14.14	NA	NA	PPP2R3A	B
rs116718733	13.94	NA	NA	PPP2R3A	B
rs111990522	NA	0.56	NA	PPP2R3A	B
rs187815576	10.16	0.5	NA	PPP2R3A	B
rs35773450	14.04	NA	NA	PPP2R3A	B
rs1393786	NA	0.75	NA	PPP2R3A	B
rs116415933	12.61	NA	NA	PPP2R3A	B
rs111611688	10.18	0.69	NA	PPP2R3A	B
rs76401139	12.63	0.5	NA	PPP2R3A	B
rs17197552	18.12	0.52	NA	PPP2R3A	B
rs9826032	20.4	0.58	NA	PPP2R3A	B
rs76760258	13.35	NA	NA	PPP2R3A	B
rs9836600	15.14	0.53	NA	PPP2R3A	B
rs6775778	11.66	NA	NA	PPP2R3A	B
rs61791718	10.42	NA	NA	PPP2R3A	B
rs113981869	10.77	NA	NA	PPP2R3A	B,E
rs2177407	17.32	0.64	NA	PPP2R3A	B,E
rs2290202	15.96	NA	NA	PRC1	B
rs17636091	10.43	NA	NA	PRC1	B
rs8031684	11.27	NA	NA	PRC1	B
rs142416384	10.06	0.52	NA	PRC1	B
rs11073964	21.9	NA	0.575	PRC1	B
rs113028686	NA	0.63	NA	PRC1	B
rs12911192	10.09	NA	NA	PRC1	B
rs16945091	10.66	NA	NA	PRC1	E
rs4932178	NA	0.85	NA	PRC1	E
